# Identification of intestinal microbial population structure of *Penaeus vannamei* supplemented with chitosan oligosaccharide using 2bRAD-M

**DOI:** 10.1128/spectrum.03148-24

**Published:** 2025-04-17

**Authors:** Chunpeng Fu, Xiaopeng Fu, Zongzhen Li, Chengjiang Xu, Fajun Li, LiXin Wang, Lifang Wang

**Affiliations:** 1 Shandong Peninsula Engineering Research Center of Comprehensive Brine Utilization, Weifang University of Science and Technology 118404, Shouguang, China; 2 Marine and Fishery Supervision Detachment of Rizhao City, Rizhao, China; University of Minnesota Twin Cities, St. Paul, Minnesota, USA

**Keywords:** 2bRAD-M sequencing, intestinal microbiome, chitosan oligosaccharide, intestinal health

## Abstract

The intestinal microbiota of *Penaeus vannamei* is critical for its health and disease resistance, but imbalances can increase the risk of infections, especially by pathogenic *Vibrio* species. This study used the 2bRAD-M method to analyze the effects of dietary chitosan oligosaccharide (COS) supplementation on the gut microbiota of *P. vannamei*. Shrimp were fed diets with COS at 5‰ and 10‰ levels. Results showed that as COS concentration increased, beneficial microbial taxa (e.g., Bacteroidetes, Actinobacteria, and *Campylobacter*) significantly increased, while harmful bacteria decreased. For example, *Vibrio parahemolyticus* abundance dropped from 0.084 in controls to 0.033 in the 5‰ group, and *Vibrio rotiforianus* decreased from 0.216 in controls to 0.121 and 0.021 in the 5‰ and 10‰ groups, respectively. *Photobacterium damselae* also declined from 0.244 in controls to 0.049 and 0.094 in the 5‰ and 10‰ groups. These findings indicate that COS supplementation can enhance beneficial microbes and reduce harmful bacteria in *P. vannamei* intestines. This study highlights COS as a sustainable dietary intervention to improve shrimp health and reduce antibiotic use in aquaculture.

**IMPORTANCE:**

This study demonstrates that dietary chitosan oligosaccharide (COS) supplementation can effectively modulate the intestinal microbiota of *Penaeus vannamei*. At 5‰ and 10‰ levels, COS increased beneficial bacteria (e.g., Bacteroidetes and Actinobacteria) while reducing harmful pathogens like *Vibrio parahaemolyticus* and *Vibrio rotiferianus*. This shift enhances nutrient absorption, immune function, and disease resistance in shrimp. COS thus offers a sustainable alternative to antibiotics in aquaculture, promoting healthy growth and reducing environmental impact.

## INTRODUCTION

The intestinal tract is the most important organ for digestion and absorption in shrimp, and it hosts a large number of microorganisms with complex structures (1). These microorganisms and the host are interdependent and mutually conditioned (2), forming a unique intestinal microecosystem. The intestinal microbial structures and compositions affect a variety of important physiological activities, such as nutrient processing (3), energy balance (4, 5), immune function (6), and growth and development of the host (7, 8). The normal intestinal flora structure enhances the immune function of shrimp and maintains the stability of the internal environment (9, 10). Beneficial bacteria can synthesize a variety of substances, participate in digestion, and decompose toxic substances (11), whereas harmful bacteria secrete toxic and harmful substances that affect the host immune system. Once shrimp develop an imbalance in their intestinal flora, harmful bacteria can invade the intestinal tract and reduce the host’s nutritional intake (12, 13).

In recent years, the rapid development of *Penaeus vannamei* culture in the world has been accompanied by the frequent occurrence of various viral and bacterial diseases. Farmers use antibiotics to deal with bacterial intestinal infections (14), but overuse of antibiotics has led to microbial resistance. This situation negatively impacts the aquaculture industry and remains a significant challenge (15). Chitosan oligosaccharide (COS), also known as β-1,4-oligosaccharide-glucosamine, is an oligosaccharide with a degree of polymerization of 2–20 degrees, which is degraded by the chitosan enzymatic process. It is the only natural sugar rich in basic amino polysaccharide. COS can be directly absorbed by intestinal epithelial cells, regulate intestinal microecology, and enhance immunity (16, 17). The antibacterial action of chito-oligosaccharides is mainly based on its polycations interacting with negatively charged groups on bacterial cell surfaces, thereby changing cell membrane fluidity and permeability, resulting in nutrient leakage and antibacterial effects (18). Additionally, COS can promote the growth of beneficial bacteria while inhibiting pathogenic bacteria (19–21).

Given the increasing demand for sustainable aquaculture practices and the need to reduce antibiotic use, understanding the effects of dietary interventions such as COS on shrimp intestinal health is of significant importance. This study aims to investigate the impact of dietary COS supplementation on the intestinal microbiota of *P. vannamei* using the novel 2bRAD-M method. By analyzing the changes in microbial community structure, we hope to provide insights into how COS can improve intestinal health and disease resistance in shrimp. This research not only contributes to the understanding of shrimp intestinal microbiota but also offers a potential alternative to antibiotics in aquaculture, thereby promoting sustainable and healthy shrimp farming practices.

## MATERIALS AND METHODS

### Sample collection

We obtained all *P. vannamei* used in this study from the same breeding pond with water temperature 38°C in Shouguang, Shandong Province, China. The prawns, with an average body length of about 11 cm, were transferred to laboratory aquariums with 30 prawns per tank. After the chitosan oligosaccharides were dissolved in water, they were evenly sprayed on the prawn feed and fed after the feed was dried. The daily feeding amount was calculated according to 5% of the body weight of the shrimp, the daily feeding frequency was four times, and the daily water change was 75%. We divided the samples into the control 0‰ (CK), 5‰ (AO1), and 10‰ (AO2) COS supplementation groups. Each group consisted of five replicates, each replicate containing 30 individuals. Samples were stored in RNA Keep-ICE Tissue Transition Buffer (Vazyme Biotech, Nanjing, China) until used for analysis.

### DNA extraction, library construction, and sequencing

DNA was extracted from tissues using a Micro DNA Kit following the manufacturer’s instructions. We prepared the 2bRAD-M library following previous research (22, 23). Briefly, the DNA (<200 ng) was digested using BcgI restriction enzyme (Thermo Fisher Scientific Inc.) at 37°C for 3 h. The digestion products were ligated to adaptors (Ada1, Ada2) overnight at 4°C T4 DNA ligase (Thermo Fisher Scientific Inc.). Subsequently, the ligation products were amplified by PCR using Phusion High Fidelity DNA polymerase (Thermo Fisher Scientific Inc.). The PCR products were purified using a QIAquick PCR purification kit and then sequenced using the Illumina Nova PE150 platform (San Diego, CA, USA).

The PE150 strategy was used to scan reads according to the identification sites of the experimental enzymes, and sequences containing enzyme fragments (called enzyme reads) were extracted. Reads containing more than 8% of N bases were filtered out, as were low-quality reads (where the number of bases with a quality value below Q30 exceeded 20% of the total number of bases in the reads). Clean reads were generated after this filtering process.

2bRAD-M sequencing was performed at Qingdao OE Biotechnology Co., Ltd (Qingdao, China).

### 2bRAD-M database construction

We obtained 173,165 microbial genomes (including bacteria, fungi, and Archaea) from the NCBI RefSeq database. We electronically digested all of them using 16 type 2b restriction enzymes to obtain unique tags under the specific taxon (not overlapping with other species under this taxon) as species-specific 2bRAD markers to generate the 2bRAD-M genome database.

### Relative abundance calculation

In the computational module of the 2bRAD-M pipeline, we employed both prebuilt and sample-specific 2bRAD marker database to perform taxonomic profiling on 2bRAD data. All sequenced 2bRAD tags were mapped to the constructed 2bRAD marker database. We calculated the G score for each species using the following formula:


$$G\;score_{species\;i}=\sqrt{S_{i}\times t_{i}}$$

where *S* is the number of reads for all 2bRAD markers mapped to species *i* in the sample, and *t* is the number of all 2bRAD markers mapped to species *i* in the sample. We screened for species with a G score >5 as candidates to control for false positives.

Subsequently, we calculated the relative abundance of each species in the sample using the following formula:


$$Relative\;abundance_{species\;i}=\frac{S_{i}/T_{i}}{\sum_{i=1}^{n}S_{i}/T_{i}}$$

where *S* is the number of reads for all 2bRAD markers mapped to species *i* in the sample, and *T* is the number of all 2bRAD markers for species in the database.

We also analyzed the composition of the two groups at the species level using linear discriminant analysis effect size (LEfSe). LEfSe is linear discriminant analysis coupled with effect size measurements. LEfSe analysis reveals the composition of different species of two or more groups of biomes.

### Statistical analysis

We used R software to conduct statistical analysis. The alpha diversity of groups CK, AO1, and AO2 was compared using the paired Wilcoxon test for Chao1 (species number and richness), the Shannon index (species richness and evenness), and the Simpson index (species diversity). We used principal coordinates analysis to calculate binary Jaccard distance and Bray-Curtis distance to statistically compare the differences in beta diversity among the three groups. Differences between any two groups were analyzed using the Kruskal-Wallis test. *P* < 0.05 was considered to be statistically significant.

## RESULTS

### Sequencing data analysis

Table 1 shows the statistics of data volume changes during sequencing quality control, including raw reads, enzyme reads, and clean reads. The average clean reads obtained by sequencing in CK, AO1, and AO2 groups were 7,774,516, 7,232,834, and 7,231,207, respectively.

**TABLE 1:** Sequencing results

Sample	Enzyme	Raw reads	Enzyme reads	Clean reads (PassQc)	Percent
AO1_1	BcgI	6,876,841	6,739,302	6,504,005	94.58%
AO1_2	BcgI	6,975,905	6,839,861	6,592,475	94.50%
AO1_3	BcgI	8,382,146	8,152,947	7,840,797	93.54%
AO1_4	BcgI	7,575,014	7,437,308	7,148,158	94.36%
AO1_5	BcgI	8,572,223	8,394,095	8,078,736	94.24%
AO2_1	BcgI	7,203,244	7,034,108	6,760,197	93.85%
AO2_2	BcgI	8,431,023	8,209,768	7,928,755	94.04%
AO2_3	BcgI	7,518,757	7,289,924	7,036,708	93.59%
AO2_4	BcgI	7,743,033	7,501,818	7,249,452	93.63%
AO2_5	BcgI	7,664,507	7,449,849	7,180,923	93.69%
CK_1	BcgI	9,069,472	8,894,973	8,551,711	94.29%
CK_2	BcgI	8,892,402	8,700,459	8,375,348	94.19%
CK_3	BcgI	6,956,049	6,824,524	6,560,353	94.31%
CK_4	BcgI	8,536,808	8,395,326	8,103,192	94.92%
CK_5	BcgI	7,719,555	7,550,318	7,281,977	94.33%

### Composition of intestinal flora community structure

**Fig 1:**
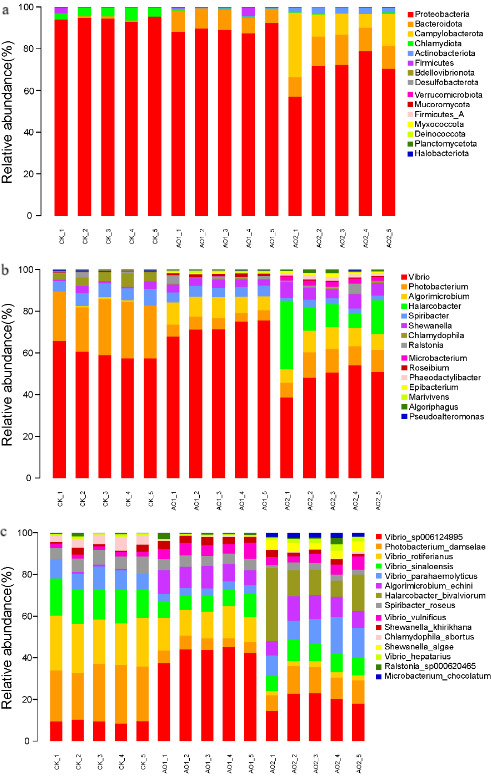
Composition of gut microbiota in each group: (**a**) relative abundance of bacterial phyla in the groups and the top 30 most abundant bacterial (**b**) genera and (**c**) species.

Figure 1a shows the composition of each experimental group at the phylum level. They consisted mainly of Proteobacteria, Bacteroidetes, Firmicutes, Tenericutes, Fusobacteria, Actinobacteria, Planctomycetes, and Verrucomicrobia. The dominant five phyla of gut microbiota in each group were Proteobacteria, Bacteroidota, Campylobacterota, Chlamydiota, and Actinobacteriota. The combined relative abundance of Proteobacteria, Bacteroidetes, and Campylobacterota was >90% in each group, but the proportion of each phylum differed. Compared with the CK group, the relative abundances of Proteobacteria (*P* = 8.17 × 10^−6^) and Chlamydiota (*P* = 2.26 × 10^−6^) were lower in the AO1 and AO2 groups, whereas those of Bacteroidota (*P* = 2.4 × 10^−7^), Campylobacterota (*P* = 0.0024), and Actinobacteriota (*P* = 1.04 × 10^−9^) were higher.

At the genus level, *Vibrio*, *Photobacterium*, and *Algorimicrobium* were predominant genera in CK, AO1, and AO2 groups (Fig. 1b). In addition, the top 5 most abundant species were *Vibrio_sp006124995*, *Photobacterium_damselae*, *Vibrio_rotiferianus*, *Vibrio_sinaloensis*, and *Vibrio_parahaemolyticus* (Fig. 1c).

### Alpha diversity

**Fig 2:**
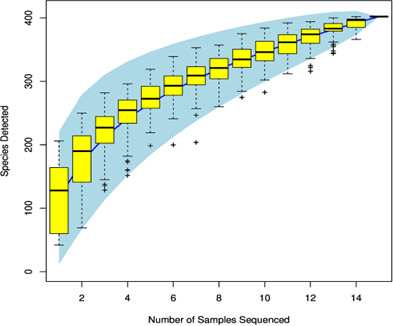
Species accumulation curves.

In diversity analysis, when the sample size is sufficiently large, the total number of species detected or the number of sequences per sample will eventually reach a plateau, where further increases in sample size result in minimal additional species detection. The point at which the curve begins to flatten indicates that the current sample size is adequate to represent the species composition of the community (Fig. 2).

The Simpson and Shannon indices are usually used to reflect the alpha diversity of bacteria in a community. A higher Shannon index value reflects higher community diversity, whereas a higher Simpson index value indicates lower community diversity. Table 2 shows that the addition of COS to the feed had a significant effect on the Shannon and Simpson index values in the intestine of *P. vannamei* (*P* < 0.05). As the amount of COS in the diet increased, the Shannon index increased from 2.24 to 2.5 to 2.93 in the CK, AO1, and AO2 groups, respectively. However, the Simpson index values of the three groups were 0.85, 0.82, and 0.90, respectively, showing a decrease in the AO1 group and an increase in the AO2 group. The Chao1 index values of the CK, AO1, and AO2 groups were 54, 119.2, and 176.6, respectively, indicating that the species richness index of the CK group was significantly lower than that of the AO1 and AO2 groups and that the difference between each pairwise comparison of two groups was significant (*P* < 0.05).

**TABLE 2:** Alpha diversity in the intestinal tract of *P. vannamei^a^*

Sample	Chao1	Shannon	Simpson
CK	54.0 ± 9.06^*a*^	2.24 ± 0.059^*a*^	0.85 ± 0.006^*a*^
AO1	119.2 ± 16.10^*b*^	2.50 ± 0.059^*b*^	0.82 ± 0.012^*b*^
AO2	176.6 ± 16.63^*c*^	2.94 ± 0.139^*c*^	0.90 ± 0.020^*c*^

^*a*^ Different letters represent significant differences (P<0.05).

Alpha diversity refers to the diversity within a specific ecosystem, and commonly used indicators include the Chao1, Shannon, ACE, and Simpson indices. Chao1 and ACE indices reflect fauna richness, with a higher value indicating greater richness (24). The Shannon and Simpson indices reflect species diversity, and a higher Shannon value means greater diversity (25). The Shannon index was derived from information entropy, and a higher value reflects greater uncertainty. In turn, greater uncertainty indicates greater biodiversity.

**Fig 3:**
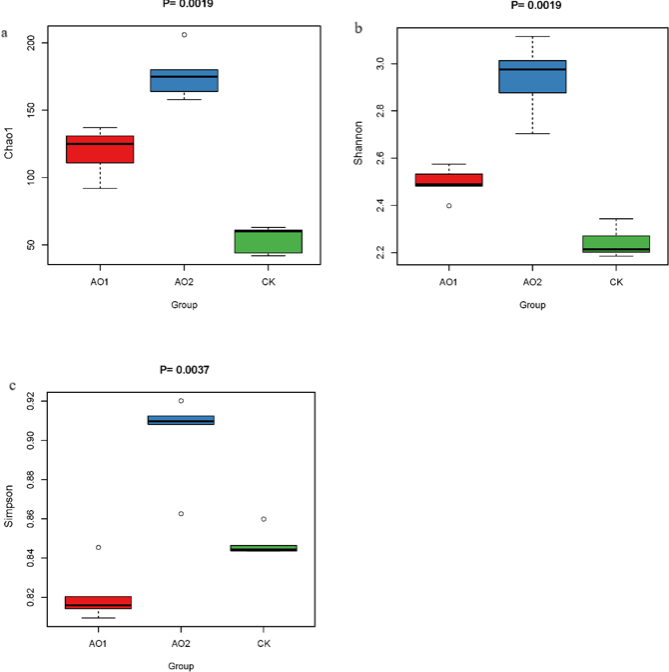
Alpha diversity: (**a**) Chao1 index, (**b**) Shannon index, and (**c**) Simpson index.

The results of our experiment showed that the Chao1 and Shannon index values increased with increasing amount of COS in the diet and that the Simpson index value decreased in the 5‰ COS group but significantly increased in the 10‰ COS group (Fig. 3).

### Beta diversity analysis

**Fig 4:**
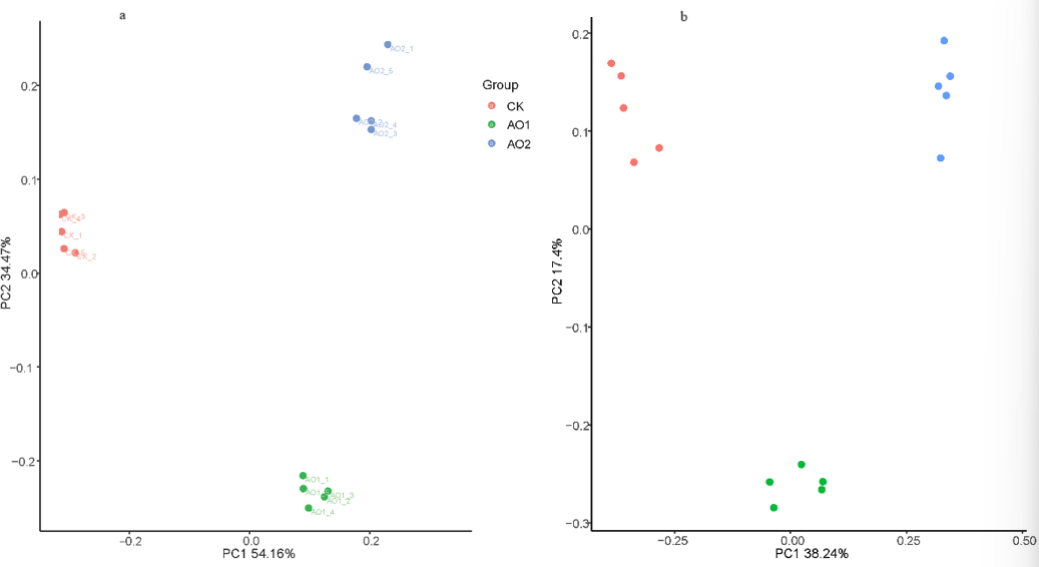
Principal component analysis normalized distribution plot: (**a**) Bray-Curtis distance and (**b**) binary Jaccard distance. Blue and green dots represent shrimp microbiomes fed with COS, while red dots represent those fed with feed that did not contain COS.

Figure 4 shows the results of principal coordinate analysis of microbial community structure in the intestine of *P. vannamei* in the CK, AO1, and AO2 groups. The Bray-Curtis (Fig. 4a) and binary Jaccard (Fig. 4b) analysis results showed significant differences in microbial community structure among the three groups (all *P* < 0.05), especially between the CK and AO2 groups (all *P* < 0.01). .The consistency in results from both distance metrics underscores the robustness of the observed variations, highlighting the significant impact of COS supplementation on the intestinal microbiota of *P. vannamei*.

### Differential abundance of microbial species

**Fig 5:**
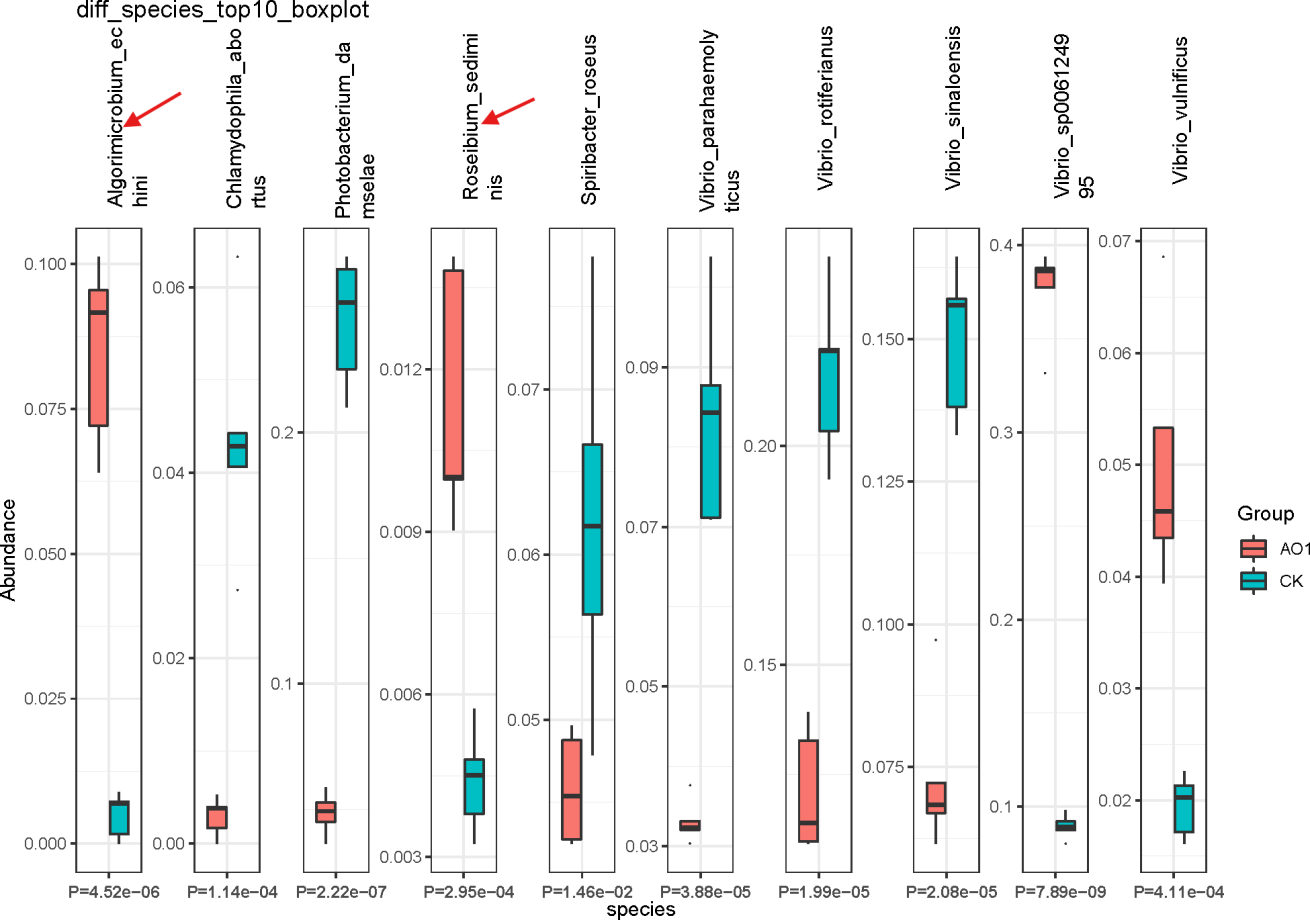
Boxplot of microbial species differential abundance between the AO1 group and CK group.

When we compared the microbial composition between the AO1 and CK groups at the species level, we found that the expression abundance of *Algorimicrobium_echini*, *Roseibium_sediminis*, *Vibrio_sp006124995*, and *Vibrio_vulnificus* was significantly different between the two groups (Fig. 5).

**Fig 6:**
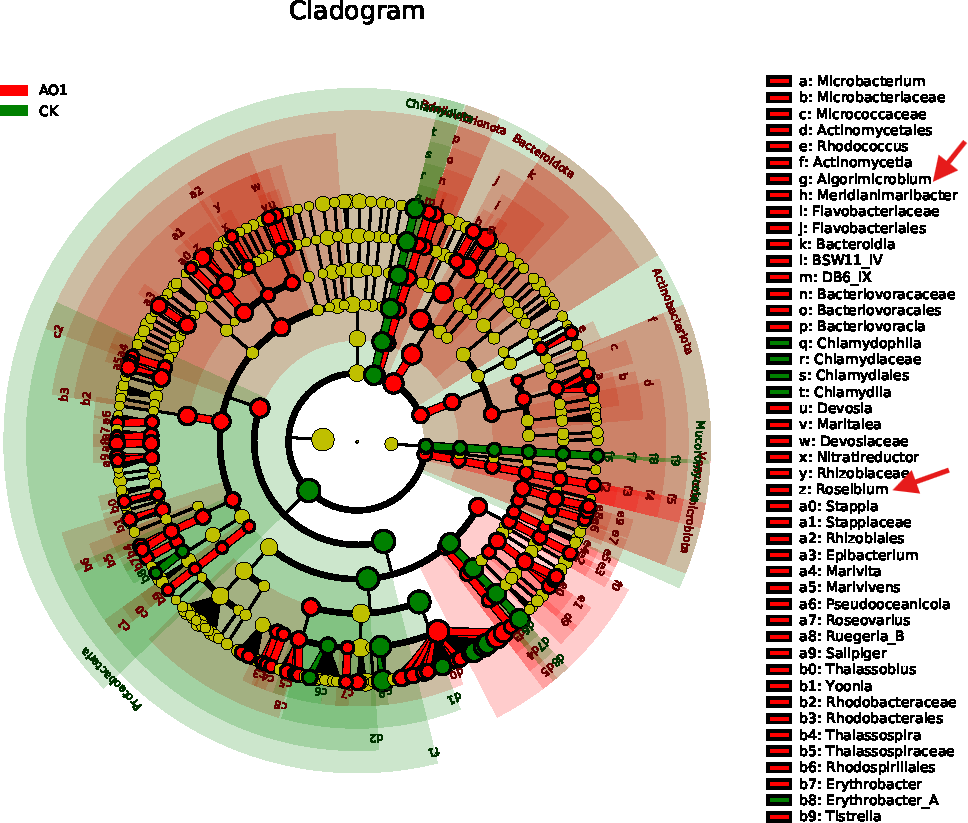
Cladogram representing the taxonomic hierarchical structure biomarkers identified by linear discriminant analysis effect size.

To further elucidate the compositional differences between the COS treatment group and the control group at the species level, we employed LEfSe. This analysis identified significant biomarkers that distinguish the microbial communities of the two groups. The cladogram in Fig. 6 illustrates the taxonomic hierarchical structure of these biomarkers.

The LEfSe analysis revealed that several beneficial microbial species were significantly enriched in the COS treatment group compared to the control group. Notably, *Algorimicrobium_echini* and *Roseibium_sediminis* were identified as key biomarkers in the COS-treated shrimp. These species are known for their potential probiotic effects, contributing to improved gut health and nutrient absorption (11, 26).

Conversely, the abundance of pathogenic *Vibrio* species, including *Vibrio_parahaemolyticus* and *Vibrio_rotiferianus*, was significantly lower in the COS treatment group. These *Vibrio* species are major pathogens in shrimp aquaculture, causing high mortality rates and significant economic losses (22). The reduction in their abundance suggests that COS supplementation can effectively mitigate the risk of bacterial infections, enhancing disease resistance in *P. vannamei*.

The cladogram provides a clear visualization of the taxonomic hierarchy of the identified biomarkers. This structure highlights the specific taxa that significantly differ between the COS treatment and control groups, emphasizing the impact of COS on the gut microbiota composition.

## DISCUSSION

### Advantages of 2bRAD-M in microbiome analysis

This study pioneers the application of the 2bRAD-M technique to characterize the intestinal microbiota of *P. vannamei*. Compared to traditional 16S rDNA sequencing, 2bRAD-M offers several distinct advantages. It provides species-level resolution and comprehensive coverage of bacterial, fungal, and archaeal communities, outperforming 16S rDNA sequencing, which is typically limited to genus-level identification (23, 27). By generating thousands of unique, evenly distributed tags across the genome, 2bRAD-M ensures superior genome coverage and minimizes false positives, even in low-biomass or degraded samples. This standardized and robust method offers high reproducibility and is highly suitable for diverse microbiome applications. Our results demonstrate its effectiveness in identifying key microbial species and their abundances, providing precise insights into the impact of COS supplementation on the shrimp gut microbiota. This technique not only enhances our understanding of low-biomass microbiomes but also establishes a powerful platform for future aquaculture research.

### Functional roles of key microbial species

The intestinal microbiota of *P. vannamei* is dominated by several phyla, including Proteobacteria, Bacteroidetes, Firmicutes, and Actinobacteria. Our study shows that COS supplementation significantly increases the abundance of Bacteroidetes and Actinobacteria while reducing the dominance of Proteobacteria. This shift is particularly noteworthy because Proteobacteria often includes many pathogenic genera, such as *Vibrio*, which are commonly associated with shrimp diseases like vibriosis (13, 28). Conversely, the increase in Actinobacteria is highly beneficial. Actinobacteria are known for their ability to produce bioactive compounds, including antibiotics and immunostimulants, which can enhance the host’s immune response and inhibit the growth of pathogens (29). Similarly, Bacteroidetes play a crucial role in carbohydrate metabolism, contributing to the breakdown of complex polysaccharides and improving nutrient absorption (30). By promoting the growth of these beneficial phyla, COS supplementation likely enhances the overall health and disease resistance of *P. vannamei*.

At the genus level, we observed a significant reduction in the abundance of *Vibrio* spp., particularly *Vibrio parahaemolyticus* and *Vibrio rotiferianus*. *Vibrio* is the most prevalent bacterium associated with diseases in crustaceans. Outbreaks of vibriosis pose a significant threat to shrimp production. These species are renowned pathogens in shrimp aquaculture, resulting in mass mortality and severe economic losses (22, 31–34). The decrease in these harmful bacteria suggests that COS supplementation can effectively mitigate the risk of bacterial infections, thereby improving intestinal health.

### Comparison with previous research

Our findings align with several previous studies on the gut microbiome of *P. vannamei*, which have consistently reported the dominance of Proteobacteria and Bacteroidetes in the shrimp gut (1, 9). However, our study uniquely demonstrates that COS supplementation can significantly modulate the gut microbiota, shifting the balance toward a more beneficial microbial community. This is in contrast to studies that have relied solely on traditional probiotics or antibiotics, which often have limited efficacy and can lead to antibiotic resistance (15, 35). Furthermore, the use of the 2bRAD-M method in our study provides higher resolution of microbial species compared to traditional sequencing techniques. For example, the significant reduction in *Photobacterium damselae* and *Vibrio rotiferianus* observed in our study has not been extensively reported in previous research, highlighting the potential of COS as a novel dietary intervention.

### Mechanistic insights into COS supplementation

The observed shifts in the microbial community can be attributed to several potential mechanisms. COS are known for their antimicrobial properties, which primarily stem from their ability to interact with negatively charged bacterial cell surfaces, disrupting membrane integrity and leading to nutrient leakage (18). This mechanism likely explains the significant reduction in harmful *Vibrio* spp. and *Photobacterium* observed in our study. Additionally, COS can promote the growth of beneficial bacteria by providing a fermentable substrate that supports their proliferation (21, 36, 37). The increase in Actinobacteria and Bacteroidetes suggests that COS may enhance the production of short-chain fatty acids and other metabolites that support gut health and immune function (26). These beneficial effects are further supported by the observed increase in alpha diversity indices, such as the Shannon index, indicating a more diverse and resilient microbial community in COS-supplemented shrimp.

### Broader implications for sustainable aquaculture

The results of our study have significant implications for the shrimp aquaculture industry. The use of COS as a dietary supplement offers a promising alternative to antibiotics, addressing the growing concern of antibiotic resistance in aquaculture (14). By enhancing the abundance of beneficial bacteria and reducing the presence of pathogens, COS supplementation can improve intestinal health, boost immune function, and ultimately increase disease resistance in *P. vannamei*. Moreover, the findings highlight the potential of COS to promote sustainable and environmentally friendly aquaculture practices. Reducing the reliance on antibiotics not only mitigates the risk of antibiotic resistance but also minimizes the environmental impact associated with antibiotic runoff. Future research should further explore the long-term effects of COS supplementation on shrimp health, growth performance, and overall productivity.

### Conclusion

In summary, our study demonstrates that dietary COS supplementation can effectively modulate the intestinal microbiota of *P. vannamei*, enhancing the abundance of beneficial bacteria and reducing the presence of harmful pathogens. These changes are likely driven by the antimicrobial properties of COS and its ability to promote the growth of beneficial bacteria. The findings provide valuable insights into the potential mechanisms underlying these effects and highlight the broader implications for sustainable shrimp aquaculture. Future research should continue to explore the specific biological mechanisms and long-term benefits of COS supplementation in various aquaculture settings.

## Data Availability

The nucleotide raw sequence reads are available under BioProject accession number: PRJNA1178095.
